# Smoking is a Risk Factor for Generation Z, Too: The Evolution of the Attitudes

**DOI:** 10.3389/ijph.2023.1604760

**Published:** 2023-02-10

**Authors:** Barbara Pavlikova, Tibor Baska, Lenka Freel, Jitse Pieter Van Dijk

**Affiliations:** ^1^ Department of Labour Law and Social Security Law, Comenius University, Bratislava, Slovakia; ^2^ Department of Public Health, Jessenius Faculty of Medicine in Martin, Comenius University, Bratislava, Slovakia; ^3^ Faculty of Medical Sciences, University Medical Center Groningen, Groningen, Netherlands; ^4^ Faculty of Medicine, University of Pavol Jozef Šafárik, Kosice, Slovakia; ^5^ Theological Faculty, Palacký University Olomouc, Olomouc, Czechia

**Keywords:** compliance, smoking, adolescents, health, GYTS, FCTC, tobacco products

## Abstract

**Objectives:** Generation Z, defined as “post-millennial,” is considered to be the first generation that could end smoking. The objective is also to take into account the evolutionary aspect of the smoking and attitudes of the Generation Z. The aim of this study was to explore the willingness of Generation Z in Slovakia to comply with the legislation adopted in the field of anti-tobacco policy and to investigate some selected social factors—intention, subjective norm and percevied behavioral control—that contribute to a lower rate of compliance.

**Methods:** Global Youth Tobacco Survey (GYTS) data on cigarette smoking among 3,557 adolescents (age range 13–15) in 2016 as well as on attitudes towards tobacco use and control measures were used to explore the level of compliance of adolescents with anti-tobacco regulations in Slovakia within the Framework Convention of Tobacco Control (FCTC). We used the concept of intention as explained in Ajzen’s theory of planned behaviour (1985), focusing on the role of subjective norm and perceived behavioural control.

**Results:** We found a decrease in ever smoking, current smoking and frequent smoking. We found that these adolescents start experimenting with dependence-causing substances, such as tobacco, regardless of existing rules.

**Conclusion:** Adolescents were attracted to smoking, although they were aware of health effects of passive smoking, and a vast majority liked smoke-free places. They are also influenced by their peers and parental models.

## Introduction

Generation Z, also defined as “post-millennials,” includes people born between the years 1995—2010. They represent approximately 32% of the world population [1]. These young people are characterized as a generation very familiar with the task of collating many sources of information and with multitasking. They are also said to be more socially aware, possibly because the Internet influences their homes, education, way of socializing and interpersonal relationships, and they see themselves as part of a global community. Compared to their parents, it is easier for them to get connected. However, they share concerns about their lives, climate change and other topics important for them [2].

Furthermore, this generation was in line to inherit a strong economy with a record low unemployment rate. However, Generation Z is also likely to self-harm, to suffer from poor body image, to skip sleep and to be overweight [3]. Their behaviours associated with poor mental health, including smoking, drug and alcohol use and vandalism, have declined [4]. They drink less and take far fewer drugs than previous generations [5]. Attention is focused on this age category because it is the period in which the first contact with the consumption of tobacco products most often occurs, and this experience, together with the factors we decided to analyze, often has an impact on whether the occasional the consumer becomes an active smoker.

More recent statistics reveal that they are much more likely to start smoking as adolescents [6]. In response, tobacco company marketing is more explicitly aimed at the youngest target group, i.e. adolescents [7]. In the World Health Organization (WHO) European Region in 2020, 1 in 7 boys and 1 in 8 girls aged 13–15 used some form of tobacco, with a total of 3.9 million adolescents being tobacco users. Promotional strategies focusing on “e-cigarettes” are particularly effective in targeting adolescents and have the potential to be drivers of long-term use of addictive and harmful nicotine products under the guise of being a healthier alternative and to sustain nicotine addiction in youth globally [8].

In this context it is relevant to ask the question of how adolescents comply with the law. The concepts of “compliance” and “non-compliance” come from international law [9–13], and we have written about this previously [14]. Several opinions on compliance are circulating [15, 16]; we adhere to those of Von Stein [17]. She understands compliance as the degree to which state (national or lower) behaviour conforms to what an agreement prescribes.

Valid epidemiological data are crucial for planning, implementing and evaluating effective preventive measures. The Global Youth Tobacco Survey (GYTS), as part of the Global Tobacco Surveillance System (GTSS), is developed by the World Health Organization (WHO) and the Centers for Disease Control and Prevention (CDC), Atlanta, USA. GYTS uses standard sampling method and uniform questionnaire providing reliably and representative epidemiologic data. This study focuses on monitoring adolescents’ tobacco use and related factors as well as key tobacco control indicators [22]. Slovakia became a part of the GTSS in 2002, and GYTS monitoring has been implemented since 2003.

The tobacco industry is a business like any other, so its main goal is to make a profit. Advertising and promoting tobacco products are its most important tools. The Framework Convention on Tobacco Control (FCTC) defines them in article 1(c) as “any form of commercial communication, recommendation or action with the aim, effect or likely effect of promoting a tobacco product or tobacco use either directly or indirectly” [18]. The majority (88%) of the 24 studies (including ten longitudinal studies) examining tobacco use conducted from 1980—2008 reported a statistically significant relationship between increased media exposure and an increase in adolescent smoking behaviour (ever having tried smoking, or age of uptake). It was noted that the evidence supporting the relationship between the media and tobacco use was stronger than that for alcohol and illicit drug use [19]. The tobacco industry spends large sums of money on advertising, sponsorship and promotion of its products. Various means of promotion are used: sports sponsorship, product innovation, campaigns targeted on women and girls, or corporate political advertising on plain packaging [20]. In Slovakia, the advertising of tobacco products is governed by Act No. 147/2001 on Advertising. This type of advertising is defined in line with the FCTC [21].

 Our research question is primarily focusing on the interconnection between TPB and smoking (by GYTS). The objective is also to take into account the evolutionary aspect of the smoking and attitudes of the Generation Z. The aim of this study was to explore the willingness of Generation Z in Slovakia to comply with the legislation adopted in the field of anti-tobacco policy and to investigate some selected social factors—intention, subjective norm and percevied behavioral control—that contribute to a lower rate of compliance.

## Methods

### Design and Samples

The GYTS consists of a school-based questionnaire study using a cross-sectional design. It employs a standardized protocol for creating samples, developing questionnaires, carrying out field procedures and processing data. A two-stage cluster sample design is used to produce representative samples of students in grades associated with the age range from 13 to 15 years (CDC—Global Tobacco Control 2018), i.e., in Slovakia grades 7–9 in primary schools and grades 2–4 in eight-year grammar schools. The sampling frame consists of all primary schools and eight-year grammar schools incorporating the above-mentioned grades (from the official list of all schools provided by the Ministry of Education). At the first sampling stage, schools are selected with a probability proportional to the school enrolment size. At the second stage, classes are randomly selected from each school included in the first stage. All students in the selected classes are eligible to participate. Student participation is voluntary and anonymous, using self-administered data collection procedures. The used methodology and its details have been described in previous publications analysing GYTS data [22–25].

In Slovakia, four waves of the GYTS have been carried out, namely in the period April—June of the years 2003, 2007, 2011 and 2016. The survey procedures were designed to protect the students’ privacy by allowing for anonymous and voluntary participation. The questionnaire was self-administered in classrooms. Students were first informed by trained field administrators about the purpose of the study and were reassured about voluntary participation and the privacy of their answers. Students recorded their responses directly into special answer sheets. During data collection, trained field administrators were present to maintain standard procedures and to provide assistance and further explanations, if needed. The surveys were approved by the Ethics Committee of the Jessenius Faculty of Medicine, Comenius University, Bratislava (EK 311/2007, EK 737/2011 and EK 1792/2016).

The basic characteristics of the samples is as follows (Supplementary Material): in 2003, the number of 13–15-year-old respondents was 3,571 (1803 boys); school response rate was 98.3%; student response rate was 87.4% and the overall response rate was 85.9% in 2003. In 2007, the number of 13–15-year-old respondents was 4,127 (2010 boys); the school response rate was 100%; the student response rate was 86.1% and the overall response rate was 86.1%. In 2011, the number of 13–15-year-old respondents was 3,888 (1912 boys); the school response rate was 100%; the student response rate was 81.8% and the overall response rate was 81.8%. In 2016, the number of 13–15-year-old respondents was 3,557 (1781 boys); the school response rate was 100%; the student response rate was 82.6% and the overall response rate was 82.6%. The overall response rate in all cases was above three-quarters of all eligible students. Drop-outs resulted mostly from student absence due to illness or other personal reasons, so they were not related to the analysed variables and could not significantly influence the results.

The further design of this study was based on the ideas of Ajzen (Theory of Planned Behaviour—TPB) as the most important starting point. We worked with publications explaining the TPB [26]. Official websites of state authorities and the texts of laws in force were also used to check for information. The relevant legislation can be found in the official Slovak Collection of Laws [27] and in the official database of EU legislation [28]. No Ethics Committee approval was necessary for this part of the documentary study.

### Measures

The uniform, internationally-based GYTS questionnaire, adapted to local conditions, was used to cover categories such as tobacco use, knowledge and attitudes regarding tobacco, environmental tobacco smoke exposure, media and advertising, desire for cessation, availability of and access to tobacco, and teaching in schools on tobacco [22, 29]. The accuracy of the translated questionnaires was checked through forward and backward translation. The following variables were defined and used in this analysis:

• Ever tried cigarette smoking, measured as the proportion of respondents who had tried or experimented with cigarette smoking, even one or two puffs.
• Current cigarette smoking, measured as the proportion of respondents reporting smoking cigarettes on one or more days in the past 30 days.
• Frequent cigarette smoking, measured as the proportion of respondents reporting smoking cigarettes on 20 or more days in the past 30 days.
• Attractiveness of smoking, measured as the proportion of respondents sharing the attitude that smoking helps people feel more comfortable at celebrations, parties and social gatherings.
• Awareness of the health effects of environmental tobacco smoke, measured as the proportion of respondents definitely thinking other people’s tobacco smoking is harmful to them.
• Positive attitude towards a smoke-free environment, measured as the proportion of respondents favouring banning smoking inside enclosed public places.

We used the TPB developed by Ajzen [26]. We measured the concept of compliance with the law through the lens of the concept of intention to comply. We measured both precursors of subjective norms through perceived social pressure and perceived behavioural control through beliefs about resources and opportunities.

The concept of (non-)compliance with the law was also crucial when searching for our scientific sources. We concentrated particularly on papers that provided us with enough information appropriate for their assessment in the light of the above-mentioned concept of TPB.

### Statistical Analyses

First, a weighing factor was applied to each student record to adjust for non-responses and variation in the probability of selection at the school, class and student levels. Then frequencies of the variables were presented as weighted percentages with the respective confidence interval of 95%. Differences between prevalence estimates were considered statistically significant if the 95% confidence intervals did not overlap.

SUDAAN, a software package for statistical analysis of correlated data [30], as well as EPI INFO were used to calculate estimates of weighted prevalence (expressed as a percentage) and 95% confidence intervals of the estimates.

### Reporting

First, we established the occurrence of smoking among adolescents. We then compiled an overview of legislation in the field of Slovak anti-tobacco policy. Next, we explored the willingness of Generation Z to comply with the officially adopted regulations in the field of that anti-tobacco policy and examined the factors that influence the higher rate of compliance using Ajzen’s TPB [26]. Then, we described intention as one of the influencing factors. Finally, we paid attention to the role of the social environment through the lens of subjective norms and perceived behavioural control, predicting that adolescents tend to engage in a certain behaviour when they evaluate it positively and when they believe that important others think they should engage in it [26].

## Results

### Occurrence of Smoking Among Adolescents

**FIGURE 1:**
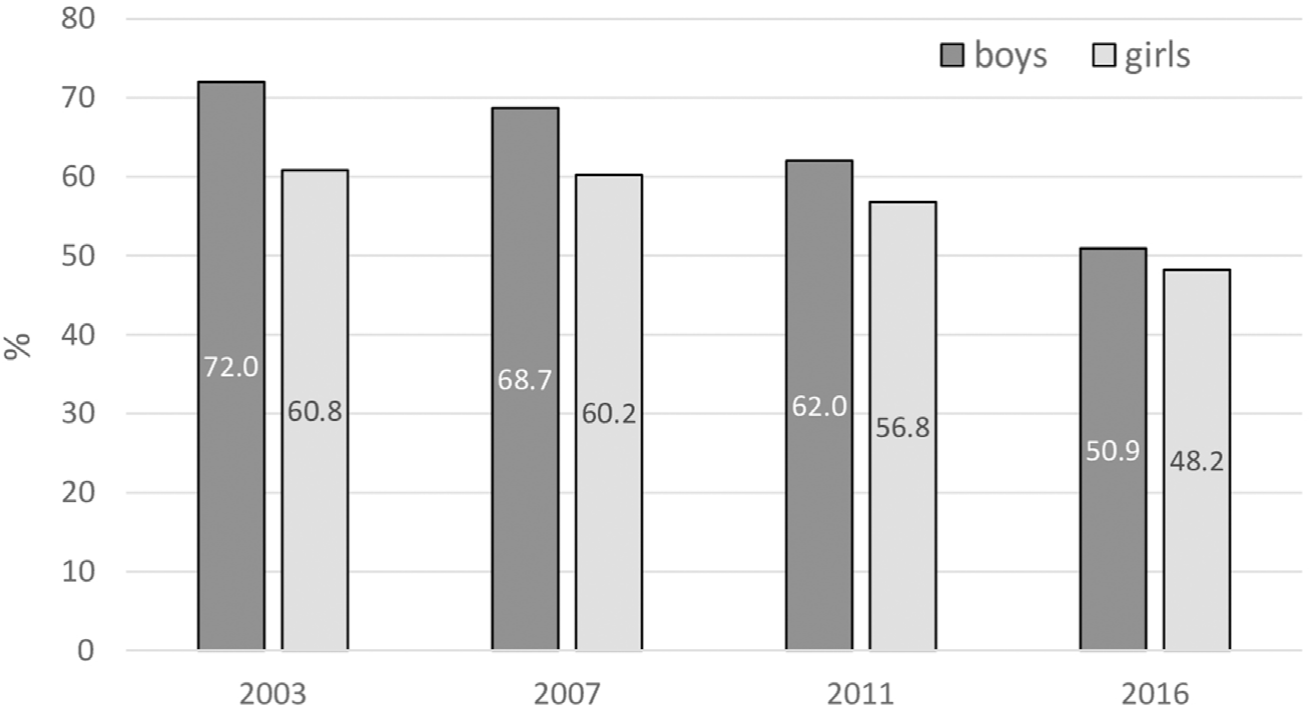
Ever tried cigarette smoking. Percentage of 13–15 years old respondents who have tried or experimented with cigarette smoking, even one or two puffs (Error bars present confidence interval 95%). (Global Youth Tobacco Survey, 2016).

Most Slovak adolescents have personal experience of cigarette smoking (Figure 1). At the beginning of the millennium, boys dominated compared with girls, but this difference has diminished in subsequent surveys, and currently no significant differences are found between the sexes. Moreover, the overall prevalence rate has gradually declined over time, mostly among boys (from 72.0% in 2003 to 50.9% in 2016).

**FIGURE 2:**
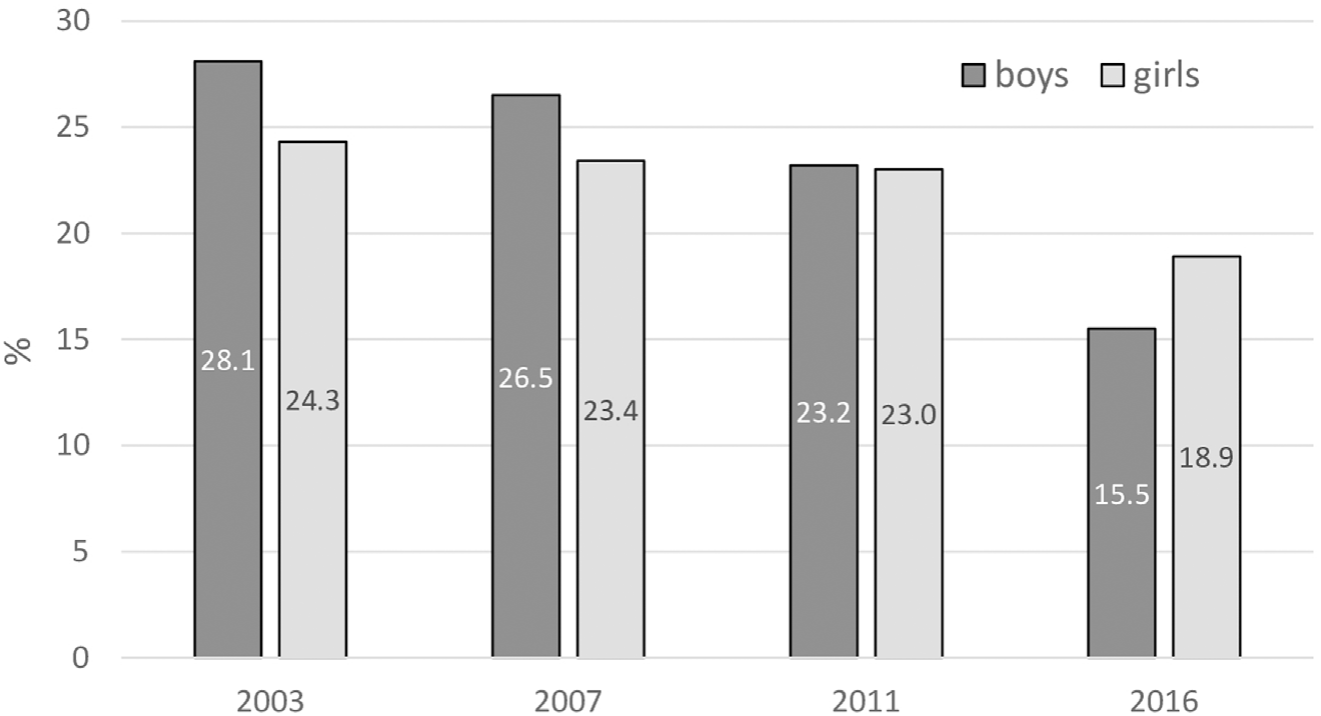
Current cigarette smoking. Percentage of 13–15 years old respondents smoking cigarettes on one or more days in the past 30 days (Error bars present confidence interval 95%). (Global Youth Tobacco Survey, 2016).

Current cigarette smoking has declined among Slovak adolescents, from over one-fourth in 2003 to less than one-fifth in 2016 (Figure 2). However, the decline achieved a significant difference only in boys (from 28.1% to 15.5%). Moreover, no remarkable differences between the sexes were seen in any GYTS survey.

**FIGURE 3:**
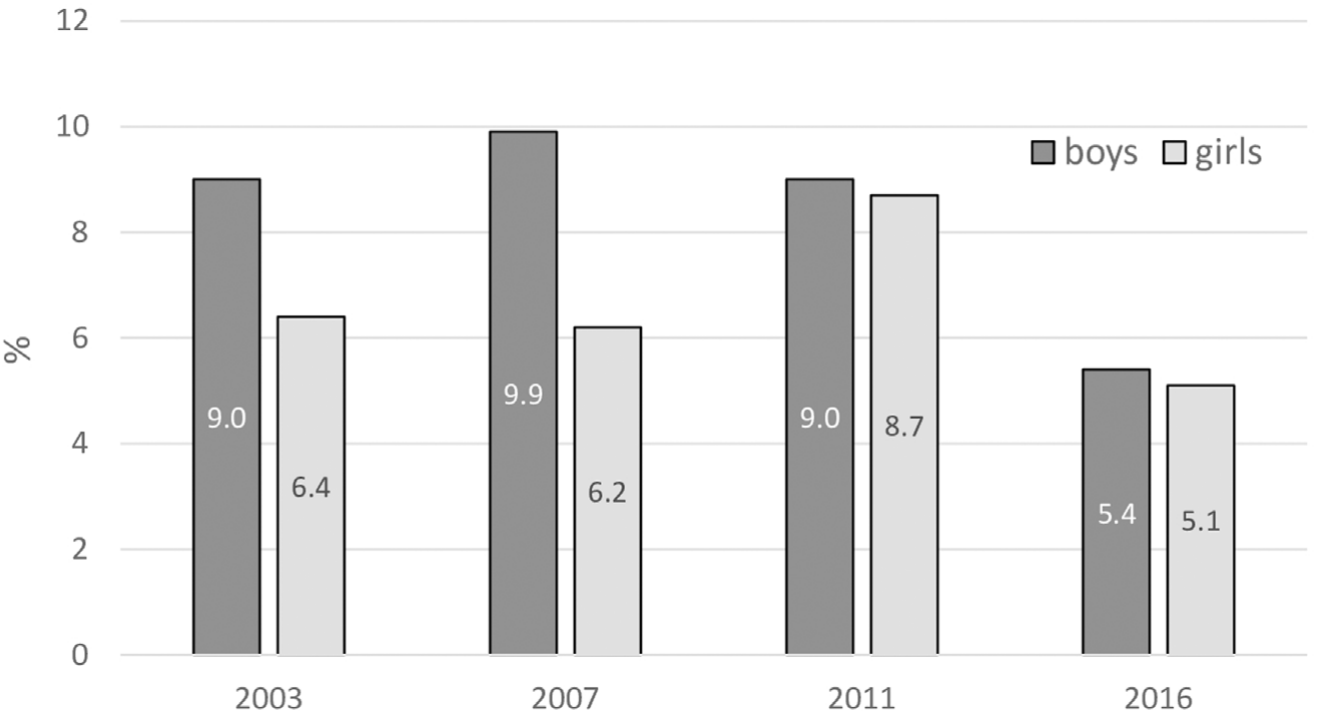
Frequent cigarette smoking. Percentage of 13–15 year old respondents smoking cigarettes on 20 or more days in the past 30 days (Error bars present confidence interval 95%). (Global Youth Tobacco Survey, 2016).

Fewer than 1 in 10 students reported smoking cigarettes 20 or more days in the last month (Figure 3). Although no remarkable statistically significant differences were found either between sexes or surveys, the findings do indicate a gradual reduction in the differences between the sexes over time due to the lower prevalence rate among boys.

### Overview of the Regulations Based on Slovakian Anti-Tobacco Policy Concerning the Promotion of Tobacco Products and the Sale of Tobacco to Minors

Slovak Act No. 377/2004 on the Protection of Non-smokers, as amended, and Act No. 106/2004 on Excise Duty on Tobacco Products, as amended, together with Act No. 147/2001 on Advertising, are the main legal instruments in the field of anti-tobacco policy. Tobacco legislation changed in 2016 and imposed new obligations on tobacco manufacturers and distributors in Slovakia. In February 2016, as a reaction to the adoption of Directive 2014/40/EU by the European Parliament and the European Council, a new amendment came into force, focusing on the size of cigarette packs [31, 32].

In its article 16 [33] the FCTC imposes an obligation on the Parties (countries who underwrote the FCTC; most countries of the world) to adopt and implement effective legislative, executive, administrative or other measures at the appropriate government level to prohibit the sale of tobacco products to persons under the age set in national law, or 18 years of age. Measures proposed by the FCTC include warnings visibly set out by sellers inside the point of sale about the prohibition of tobacco sales to minors (<18); requesting appropriate evidence from buyers of having reached full legal age (≥18); banning the sale of tobacco products in directly accessible manner; and prohibiting the manufacture and sale of sweets, snacks, toys or any other objects in the form of tobacco products. Public promotion of free tobacco products and the sale of cigarettes individually or in small packs are also prohibited.

Slovak Act No. 377/2004 on the Protection of Non-smokers deals with these obligations in section 6 [34]; it states that the sale of tobacco products and products intended for smoking and not containing tobacco is prohibited in the case of minors and also in places where they are easily accessible for people under eighteen. Act No. 147/2001 on Advertising regulates the ban on advertising tobacco products and exceptions to it in section 6 [21].

### Social Environment and Compliance With Anti-Tobacco Regulations

**FIGURE 4:**
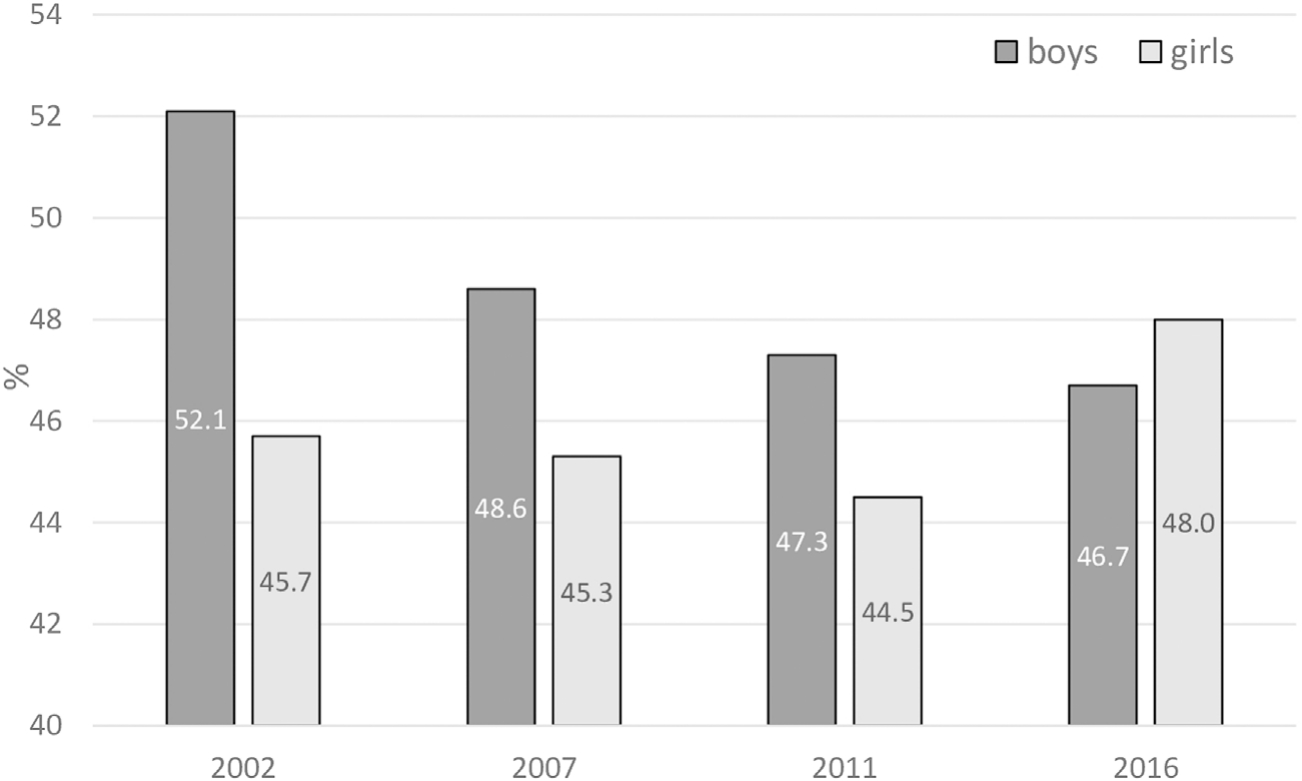
Attractiveness of smoking. Percentage of 13–15 years old as a proportion of respondents sharing the attitude that smoking helps people feel more comfortable at celebrations, parties and social gatherings (Error bars present confidence interval 95%). (Global Youth Tobacco Survey, 2016).

About one-half of all respondents considered smoking attractive, sharing the view that this behaviour helps people feel more comfortable at celebrations, parties and social gatherings (Figure 4). Boys significantly dominated compared to girls only in 2002, while in later surveys differences between the sexes were slight, not achieving statistical significance. Moreover, the approval rates remained similar throughout the analysed period.

However, adolescents are less compliant with health laws when they are surrounded by their peers and they need to feel better (Figure 4). This is not only the influence of social pressure, but also of their individual inner expectations and feelings.

**FIGURE 5:**
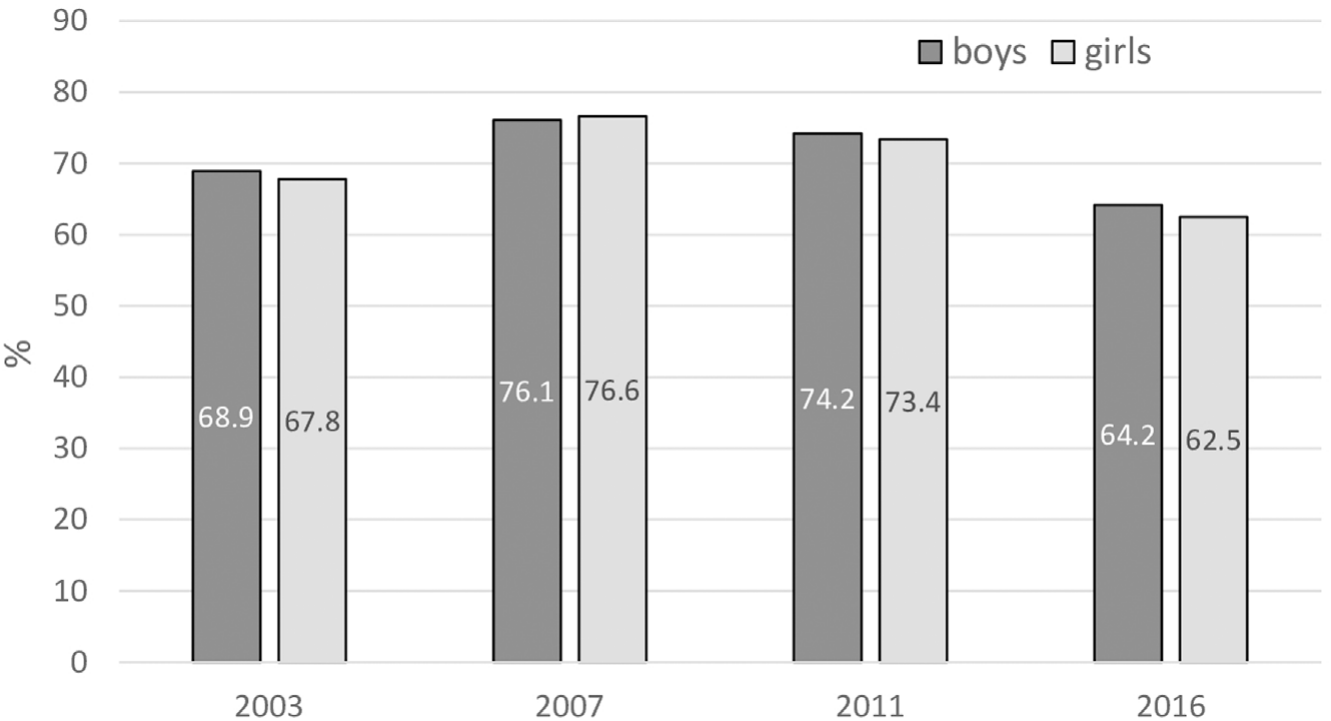
Awareness of health effects of passive smoking. Percentage of 13–15 year olds as a proportion of respondents definitely thinking other people’s tobacco smoking was harmful to them (Error bars present confidence interval 95%). (Global Youth Tobacco Survey, 2016).

Most of the respondents held the view that other people’s tobacco smoking was harmful to them, maintaining almost the same rates in boys and girls (Figure 5). The percentage significantly increased between 2003 and 2007 but later decreased in 2016 to values very similar to 2003.

**FIGURE 6:**
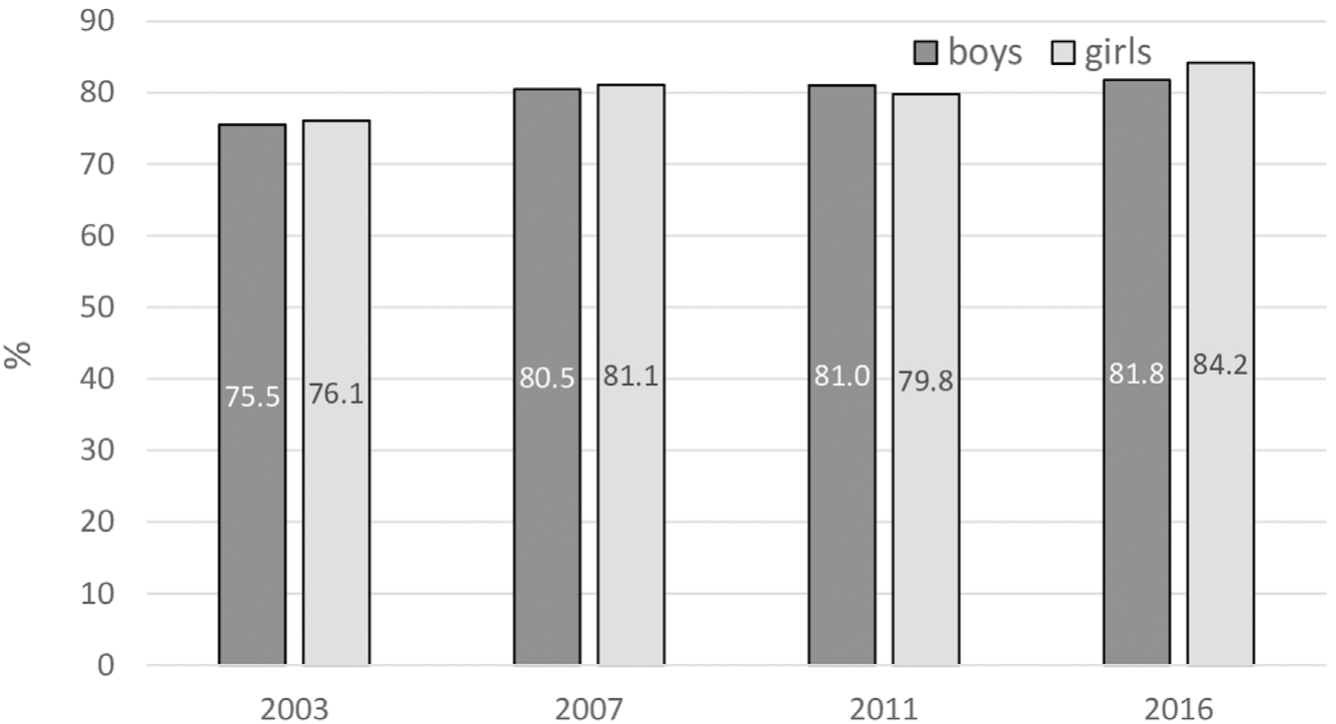
Positive attitudes towards a smoke-free environment. Percentage of 13–15 years old as a proportion of respondents favouring the banning of smoking inside enclosed public places (Error bars present confidence interval 95%). (Global Youth Tobacco Survey, 2016).

An overwhelming majority of students, similarly in boys and girls, favoured banning smoking inside enclosed public places (Figure 6). The percentage gradually increased from about three-quarters in 2003 to more than 8 out of 10 in 2016.

## Discussion

We explored the willingness of Generation Z in Slovakia to comply with the regulations officially adopted in the field of anti-tobacco policy in line with the FCTC, and we investigated selected social factors contributing to a lower rate of compliance. When it comes to compliance with the law, Generation Z appears to be influenced by certain social factors: young people are more open to smoking in relation to various social gatherings, such as parties, although they are aware of the harmful effects of tobacco use, regardless of their knowledge of legal regulations.

### Occurrence of Smoking Among Adolescents

The GYTS Slovakia findings show that tobacco use remains very popular among adolescents, since the majority of them reported personal experience with cigarette smoking. As Fidler et al. [35] present, there was also a correlation between the first smoking experience and the later tendency to smoke. Their study concludes that preventing children from trying even one cigarette may be important. More than half of first-cigarette smokers continue to smoke on a limited but regular basis, i.e. at least one cigarette a month, and a considerable proportion of them smoke frequently, which indicates a high probability of development of addiction and presumptive long-term tobacco use in adulthood leading to consequent health effects [36].

The findings indicate—contrastingly—a declining trend, particularly since 2011, especially remarkable among boys. This development is gradually leading to the disappearance of the traditional predominance of males, although this is still present in the adult Slovak population [37].

This situation corresponds to the overall decline in substance use in Europe [38]. However, we should be careful about considering this development too optimistically. The emergence of new, more exotic forms of tobacco use (water pipes, electronic cigarettes) among adolescents during recent years is also a reason to be careful [39–41], because it indicates that one of the possible reasons for the decline in cigarette smoking is the increased attractiveness of other products instead of cigarettes. A contrasting development is the “smoke-free generation,” a successful grassroots movement among a lot of organizations (among which hospitals, municipalities, universities) in the Netherlands.

### Regulations in the Slovak Anti-Tobacco Policy Regarding the Promotion of Tobacco Products and the Sale of Tobacco to Minors

We further found that Slovakia’s legal regulation of selling tobacco products to minors and the regulation of advertising tobacco products is in line with the FCTC. The set rules are clear and are the subject of state control. Violating them is punished in accordance with the applicable legislation. Our finding is consistent with other studies [42, 43], which have already demonstrated the dependent relationship between adolescent smoking and promotion of tobacco products. The relationship between tobacco marketing and smoking among youngsters exposed to cigarette advertising is associated with smoking behaviour and the intention to smoke [44]. Higher levels of advertising and greater visibility of cigarette promotions are associated with smoking uptake [45]. All Parties to the FCTC are required to implement the ban on selling tobacco products to minors in their legislation. Youth smoking prevention and control efforts have had mixed results, and research shows that combining various strategies can lead to clear results. Aggressive media campaigns, teen smoking cessation programmes, social environment changes, community interventions, and increasing cigarette prices could be useful tools [46] in supporting the positive tendency in smoking rates among adolescents in Slovakia. According to our opinion, -well-thought-out regulation of tobacco promotion and appropriate, enforceable sanctions can lead to reduction in smoking in that age group.

### Social Environment and Compliance With Tobacco Control Measures

As the GYTS Slovakia findings show, about half of adolescents in Slovakia consider regular smoking as an effective social mediator. We should therefore consider this attitude as a common motivational factor promoting tobacco use in this age group. On the other hand, the overwhelming majority of them are aware of the dangers of passive smoking and share positive views towards smoke-free control measures. However, the trends in these two indicators are different. While awareness of the dangers of passive smoking has decreased noticeably since 2011, positive views towards smoke-free measures have been gradually increasing. This apparently inconsistent development can be explained in terms of reduced awareness of the topicality of smoking as its prevalence has declined. On the other hand, as smoking plays a less important role among adolescents, they do not perceive restrictions against it as affecting their activities [47].

### The Role of Subjective Norms and Perceived Behavioural Control in Compliance

Finally, the risk of smoking among adolescents is influenced by the incidence of smoking in their surrounding social environment. Teens go through great developmental changes, forming their own identities and taking more risks than their elders. Cigarette-smoking may make teens feel popular, sophisticated, attractive or tough [48], and as smokers, they feel more comfortable in social gatherings. This trend can be seen among both sexes. Our findings indicate that perceived social pressure to behave in some manner (subjective norm) often leads to behaviour expected by the social environment with the strongest influence, regardless of its harmful effects. In practice, youngsters tend to find ways of bypassing existing obstacles resulting from legal regulation, most often through older friends who influence their perceived behavioural control, which again leads to non-desirable behaviour (smoking). However, as the declining trend among boys indicates, the influence of the social environment can also work in the opposite direction. This finding supports the idea of implementation of campaigns and preventive measures mainly aimed at reinforcing awareness of the harmful effects of active and passive smoking in a non-violent way and in connection with social events. Our findings indicate that youngsters are aware of the harmful effects of not only active but also passive smoking (Figure 5), and they appreciate the growth of smoke-free environments (Figure 6). It appears to be necessary to show these adolescents different ways by which they can make themselves feel more comfortable when they are part of the social environment and to pre-empt their combining smoking with a sense of release.

### Strengths and Limitations

Our study aimed to explore how the adolescents called “Generation Z” behave in the area of compliance with health laws, and which factors are most influential in terms of their decision to take up smoking or not. First, we described the occurrence of smoking among schoolchildren using a large representative sample. The survey questions were those previously used in the GYTS Slovakia study. Then, we considered the analysis of this issue through the lens of Ajzen’s TPB. This approach enabled us to find a new perspective when it came to searching for the reasons for smoking. We have no information about other studies carried out in the field of non-compliance which apply the concept of selected aspects of the TPB in other age groups, and we regard this as the one of the strengths of this work. The small number of studies dealing with the issue of adolescent approaches to smoking from various points of view in general is one of the limitations of our study. Furthermore, we can mention some limitations related to the TPB, such as the assumption that behaviour is the result of a momentary decision-making process, ignoring the possibility that it can change according to inner or external circumstances, or that it involves attention to environmental and economic factors.

### Implications

Our findings confirm the significance of group rules. This supports the idea that community-level health-promotion intervention programmes can be an effective tool with a real impact on the current and future health of youngsters, but only if applied properly.

We still need more relevant and demand-oriented research in this area to be able to provide evidence-based recommendations for policy and practice. As part of the general effort to reduce smoking among adolescents, the most effective ways of really influencing older members of that age group need to be addressed. Research should also be done in the field of the impact of new social media (Instagram, TikTok, Clubhouse) on the lifestyle of adolescents and the possibilities of their application in the process of building awareness of healthier living in this age group. Research into the application of a participatory approach in this age group also shows interesting potential. We need to look for opportunities to influence youngsters through the attitudes of other young people.

### Conclusion

The GYTS Slovakia study revealed that smoking has a decreasing trend among boys. The vast majority of respondents perceive the legislation to limit smoking positively. It further shows that attitudes towards smoking do not change during the observed period. Changes in smoking indicators do not correspond to changes in legislation, this indicates the importance of other social factors.
