# Supplementary figures and images for: Smoking is a Risk Factor for Generation Z, Too: The Evolution of the Attitudes
Source: Int J Public Health. 2023 Feb 10;68:1604760. doi: 10.3389/ijph.2023.1604760 (PMC9971969; doi:10.3389/ijph.2023.1604760)

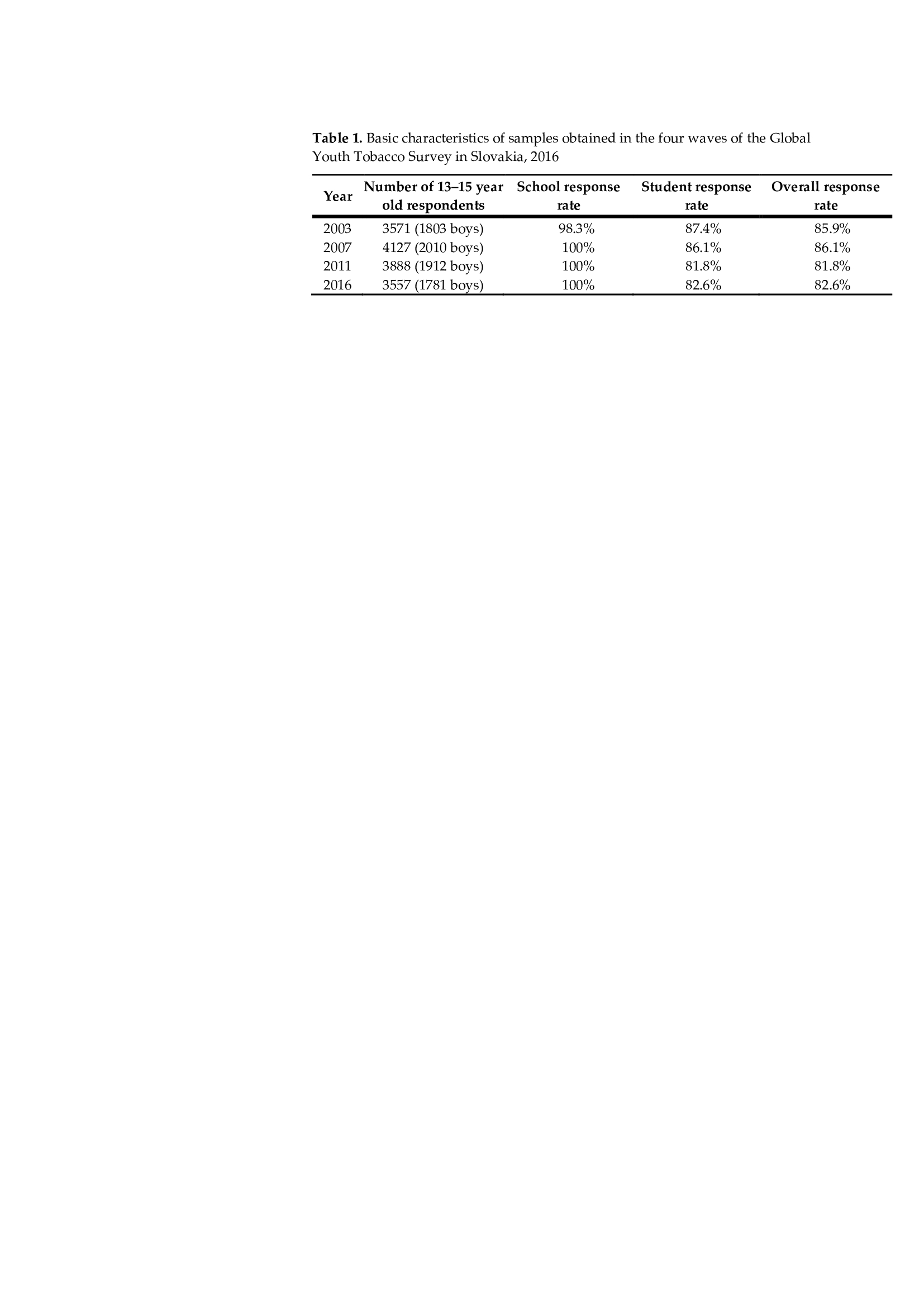

Supplement: Supplementary file 1 [file Image1.JPEG]
